# Application of Hydrogels in the Device of Ophthalmic Iontophoresis: Theory, Developments and Perspectives

**DOI:** 10.3390/gels9070519

**Published:** 2023-06-26

**Authors:** Dong Wei, Ning Pu, Si-Yu Li, Na Zhao, Zong-Ming Song, Ye Tao

**Affiliations:** 1 Henan Eye Institute, Henan Eye Hospital, Henan Provincial People’s Hospital (People’s Hospital of Zhengzhou University), Zhengzhou 450003, China; 2 College of Medicine, Zhengzhou University, Zhengzhou 450001, China

**Keywords:** hydrogel, iontophoresis, ocular, drug delivery

## Abstract

The human eye is a consolidated organ with delicate structures and unique immune privileges. Ocular diseases are intractable due to the intrinsic biological barriers within the eyeball. Hydrogels are excellent drug-carrying substances with soft material and excellent properties. They have been extensively used to deliver drugs into ocular tissue via iontophoresis devices. Ophthalmic iontophoresis is an electrochemical technique using tiny electrical currents to deliver drugs into the eye non-invasively. The early infantile iontophoresis technique often required long applying time to achieve therapeutic dose in the posterior ocular segment. The potential limitations in the initial drug concentration and the maximum safe currents would also impede the efficiency and safety of iontophoresis. Moreover, the poor patient compliance always leads to mechanical damage to the cornea and sclera during application. Advantageously, the flexible drug-carrying hydrogel can be in direct contact with the eye during iontophoresis, thereby reducing mechanical damage to the ocular surface. Moreover, the water absorption and adjustable permeability of hydrogels can reduce the electrochemical (EC) reactions and enhance the efficiency of iontophoresis. In this review, we focus on recent developments of hydrogels iontophoresis in ophthalmologic practice. Refinements of the knowledge would provide an outlook for future application of hydrogels in treating ocular disease.

## 1. Introduction

The human eye is a consolidated organ with unique anatomical architectures and physiological characteristics [1]. Several complicated biological barriers make it very challenging to deliver drugs to the lesion within the eyeball. Thus far, the commonly used methods of ophthalmic drug delivery, such as topical drug delivery, systemic drug delivery, vitreous cavity injection, and change of drug dosage or structure, have their own drawbacks [2,3]. Delivering drugs to the eye non-invasively remains an important problem in clinical practice. Notably, the iontophoresis technique, which relies on tiny electric currents for delivering drug to the lesion, has been invented by scientists. Thus far, this technique has been used in a wide range of medical experiments and clinical trials. However, researchers realize gradually that some drawbacks are implicated in the iontophoresis technique. If the applied current of the iontophoresis device is too small, the initial drug dosage fails to attain the effective therapeutic concentration within the ocular tissue. On the other hand, if the applied current is too large, it will induce EC reaction to the cornea and sclera [4,5]. In order to enhance the delivery efficiency and relieve potential mechanical damage caused by conventional devices, novel hydrogels with control release capacity have been developed for the iontophoresis technique (Table 1). These hydrogels can alleviate EC reaction during iontophoresis, and mitigate pH changes within the ocular tissue [5,6]. Therefore, hydrogel is an essential component of the advanced iontophoresis devices in future clinical practice.

## 2. Physiological and Anatomical Barriers Facing Ocular Drug Delivery

Eye diseases always cause visual impairments and will affect the quality of daily life. However, it is difficult to deliver therapeutic drugs into the eyeball because this organ is a pharmacological sanctuary. The anterior segment, mainly the cornea, is composed of avascular tissue and its relative transparency makes this architecture responsible for three-fourths of the total refractive power of the eyeball [7]. Precorneal dynamic barriers, including the aqueous tear film, the mucin layer, and the mucus layer will be responsible for a loss of 95% of topically administered drugs [14]. Another dynamic barrier is the presence of transmembrane efflux pumps in the corneal surface, which also restricts drug transport across the cornea [15]. Furthermore, when the residual drug molecules are in contact with the corneal surface, they will be hindered by the corneal epithelial barrier. Corneal epithelial cells are fixed together to form a complex network of connections. These tight intercellular junctions are distributed at different depths of the stratified epithelia, resulting in a strong resistance barrier against drug absorption [16]. Alternatively, the drug is delivered through the sclera pathway, which can bypass the cornea barriers. However, the challenges of static, dynamic, and metabolic barriers should also be addressed properly. Static barriers are ocular structures that hinder drug penetration through their own biological tissues, including the conjunctiva, sclera, choroid, Bruch’s membrane, and retina [17]. When drug molecules cross the sclera tissue, the choroidal vascular network, lying between the retina and the sclera, can also serve as the static barrier against the drug diffusion [18]. The dynamic barrier is formed during the clearance of drugs through blood and lymphatic vessels, as well as during drainage of bulk fluid through the eye [19]. For instance, the drug molecules on the sclera surface will be absorbed into the bloodstream through the episcleral vein, and then they are cleared by the conjunctival lymphatic system. Furthermore, the blood retinal barrier (BRB) formed by the retinal pigment epithelial (RPE) and retinal vascular endothelium acts as a huge obstacle against drug delivery [20]. PER et al. found that large molecules injected into the system circulation could escape from the walls of the choriocapillaris and penetrated the Bruch’s membrane [21]. However, these molecules are eventually blocked by the connection of RPE. Additionally, the blood-aqueous barrier (BAB) located in the anterior segment is also recognized as another component of the biological barrier system. BAB is composed of tight junctions in the ciliary nonpigmented epithelium, the endothelial cells in the iris vasculature, and the inner wall endothelium of Schlemm’s canal [22]. Ciliary body and RPE are the most active sites for xenobiotic metabolism in the eyes, since they are responsible for detoxifying drugs delivered through systemic circulation. The efficiency of transscleral delivery may be limited by the degrading and detoxifying enzymes in the ciliary body and RPE [19]. In this context, systemically administered drugs cannot cross the BRB or BAB, and reach the posterior segment of the eye [23]. Similarly, these drugs are unable to reach the posterior segment via the topical route due to the low corneal epithelial permeability, systemic absorption, and these biological barriers. Although intravitreal injection can attain the maximum drug concentration in the vitreous cavity, various complications may be caused by this invasive surgery, including cataract, glaucoma, choroidal hemorrhage, endophthalmitis, vitreous hemorrhage, retinal rupture, and retinal detachment [24]. Therefore, improving transport efficiency through non-invasive technique is important in the treatment of ocular diseases.

## 3. Theory and Development of Iontophoresis

**Figure 1:**
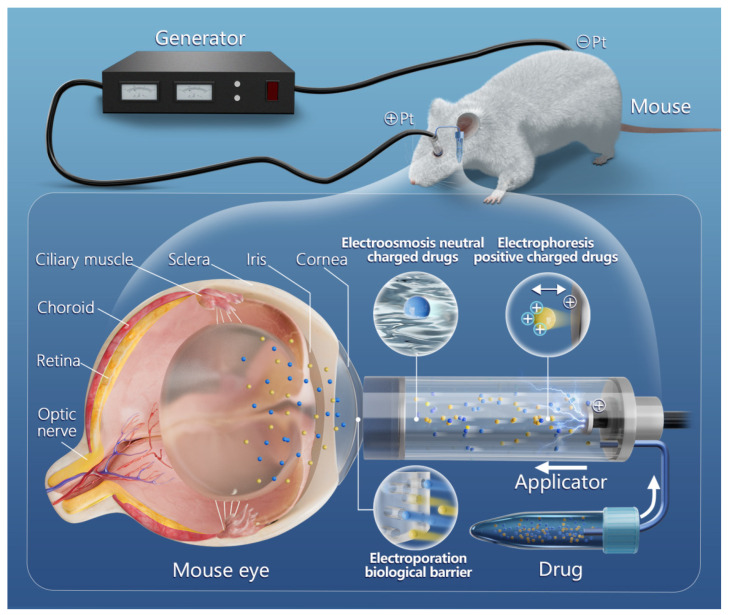
The basic principle of transcorneal iontophoresis transporting electrically neutral and charged drugs (taking positively charged drugs as an example).

As early as the Golden Age of the Greek civilization in ancient times, Pivati proposed the idea of using an electric current to increase the penetration of charged drugs into biological tissues [25]. In the past decades, the iontophoresis technique has developed rapidly and has been used widely to deliver drug molecules for the treatment of eye diseases, including inherited retinal degeneration, dry eye syndrome, and pseudomonal keratitis [26,27,28]. Iontophoresis is defined as the process of passing ionized drugs through biological membranes under the action of an electric current. It is a non-invasive technique of transporting ionic or charged molecules from electrolyte solutions to biological tissues through direct or periodic currents [29,30]. Iontophoresis is based on the principle that oppositely charged ions attract each other, while the same charged ions repel each other under the control of electrode polarity (Figure 1). Electrorepulsion drives the ionized drugs into the tissue from the cathode (for positive drugs) or anode (for negatively charged drugs). It is the ionic-electric field interaction, called electrophoresis (direct-field effect) or the Nersnt-Planck effect, that dominates the flux enhancement of small ions [30]. In addition to the direct interaction between the electric field and the charge of ionic penetrant, there are two other mechanisms responsible for the enhanced drug delivery: electroosmosis, and electroporation [31]. Electroosmosis is mainly in charge of the passage of ionic and neutral drugs. This mechanism helps to transport neutral and charged molecules along the bulk solvent flow generated by the electric field [32]. On the other hand, electroporation, also known as electro-permeabilization, seek to change the tissue barrier by increasing the intrinsic permeability (e.g., membrane porosity) and generating the permeability path under the influence of electric field (e.g., membrane pore charges and sizes). During the process of electroporation, some aqueous channels are formed within the lipid bilayer of membrane. According to the size and duration of the applied electric field, the aqueous channels are endowed with transient, reversible, or irreversible features [32,33,34]. Iontophoretic drug delivery is generally considered safe and mild, as the potential gradient drives molecular transport across the membrane. Since iontophoresis primarily drives drug molecules through ionic-electric field interaction rather than disturbing eye tissue, it will not cause invasive eye damages [35]. The resulted drug level by iontophoresis is significantly higher than that by passive delivery. Therefore, iontophoresis can reach the therapeutic concentration faster. In addition, if necessary, the drug concentration in the eyeball can be adjusted by altering the current intensity and apply duration [4,10]. Hitherto, three types of patented iontophoresis devices, coulomb controlled iontophoresis (CCI), EyeGate II delivery system (EGDS), and hydrogel ionic circuit (HIC) have been extensively used to deliver drugs into eyeball.

## 4. Application of Traditional Iontophoresis Device in Ophthalmic Practice

### 4.1. CCI

The CCI device is composed of a generator, a pump to drive the drug, a pair of electrodes, and an eye cup [36]. CCI is the first generation of iontophoresis device that has been widely used in ophthalmic experiments and clinical trials. In 1997, Behar-Cohen et al. used the CCI system to deliver dexamethasone (Dex) to the uvea of an endotoxin-induced uveitis model [36]. To ensure the safety and efficiency of the CCI system, they used a tiny current of 400 μA, and each iontophoresis lasted for 4 min. The experimental results showed that the therapeutic effect of the iontophoresis group using a current density of 1.2 mA cm^−2^ was much more superior compared with the intraperitoneal Dex injection group. These findings indicate that iontophoretic delivery of Dex can act as an alternative strategy of systemic glucocorticoid administration in the treatment of acute uveitis [36]. Moreover, Hayden et al. used the CCI system to deliver carboplatin into the eyes of mice with retinoblastoma [37]. They treated 30 LH_BETA_T_AG_ mice (in short, the transgenic mice were inserted with a gene that highly expresses SV40 large T antigen to induce bilateral retinoblastoma) with iontophoresis twice a week. The current density was maintained at 2.57 mA cm^−2^ and each treatment lasted for 5 min. The results showed that the therapeutic effect depended on the carboplatin concentration in the retinoblastoma lesion. Iontophoresis treatment with a carboplatin concentration of 7 mg/mL was the most effective dosage, with 50% (4/8) of retinoblastomas being controlled. Safety profiles suggested that carboplatin concentrations lower than 10 mg/mL did not induce any toxic effects to the cornea. Therefore, it is possible to raise the therapeutic effects not only by increasing the carboplatin concentration in the CCI eye cup, but also by enhancing the current of iontophoresis. However, the CCI device does not achieve a more satisfactory result due to the strict limitation of applied current [37]. In this context, many research groups have evaluated the safety and efficacy of drug delivery by CCI in ophthalmologic practice [4,38,39,40,41]. The results showed that a current density of 2 mA cm^−2^ is generally safe and effective for the CCI device in the treatment of anterior segmental diseases [40]. For posterior segment diseases, transscleral iontophoresis is generally safer with a current density of 5 mA cm^−2^ [38,39,41]. Although these findings may ensure the safety of the CCI device to some extent, the efficiency of iontophoresis is significantly reduced. Therefore, it is imperative to update the iontophoresis device, and to up-regulate the maximum safe current as soon as possible. These reformations will pave the path for further application of iontophoresis technique in clinic work.

### 4.2. EGDS

EGDS (EyeGate Pharmaceuticals, Inc., Waltham, MA, USA) is an iontophoresis device with full usage criteria assembled on the basis of CCI. It has the same constituent parts and configurations as CCI. However, its controller is more compact and convenient, and the current produced by the power battery is more stable and safer. Based on experimental data from CCI, the EGDS has been used for treating ophthalmic disease in clinical trials [42,43,44]. In 2011, Patnae et al. designed an EGDS device to deliver EGP-437 (dexamethasone phosphate, DP) for the treatment of dry eye symptom [42]. Patients with definite dry eye disease were randomized into three groups (DP7.5 group: 7.5 mA-min at 2.5 mA, *n* = 41; DP10.5 group: 10.5 mA-min at 3.5 mA, *n* = 37; placebo group: 10.5 mA-min at 3.5 mA, *n* = 25). The combined outcome of the patients in the DP7.5 and DP10.5 groups showed significant improvements in terms of clinical signs and symptoms compared with placebo group. In greater detail, the prognosis in the DP7.5 group was better compared with the DP10.5 group, indicating that the low-current treatment was more efficient for delivering the drug to the anterior segment of eyeball. For safety, acute events (AEs) occurred in nine patients after iontophoresis, and all AEs associated with iontophoresis resolved within 24 h [42]. Two other similar clinical trials have also used the iontophoresis to deliver DP to treat non-infectious uveitis. They used anterior chamber cell (ACC) scores to assess the efficacy of iontophoresis. The results showed that iontophoresis of DP was more effective to treat the patients with severe uveitis. The trials agreed well with each other, and no serious complications occurred in either clinical trial [43,44].

## 5. Drawbacks of Conventional Iontophoresis Devices

At the initial development stages, iontophoresis technology lacked standardized parameters for clinical application. The pioneering iontophoresis devices cannot precisely control the current density, application time, and rate of drug permeation. Consequently, some potential toxic and side effects might be caused by iontophoresis. As early as 1986, Michael Balza et al. applied a 10 min transscleral iontophoresis (with the current density of 200 amps/cm^2^) using an ion permeation device. The transscleral iontophoresis caused focal corneal damage at the applied site, while corneal iontophoresis had lower risk of inducing corneal damage [45]. With the accumulation of in-depth evidence, more toxic effects of iontophoresis gradually emerged. For instance, transscleral iontophoresis can result in the attenuation and disorganization of retinal layers [41]. Once the ocular tissue is damaged by heat, altered hydration, and/or mechanical disorganization during iontophoresis, their impedance will change over time, resulting in variable electrical fields (volts per square centimeter). These electrical alterations can easily occur at the epithelial surface (e.g., conjunctiva), especially when high current densities are applied [41,46]. In addition, iontophoresis through the cornea has also been reported to cause corneal epithelial edema and reduced endothelial cell count, corneal opacity, and interstitial edema [6,47,48]. The change of pH at the contact interface between the current device and the ocular tissue is another risk factor. In the contact area between the current device and the ocular tissue, the electron flow will be converted into an ion flow. When the current intensity increases during this process, water electrolysis will occur and induce local pH changes [5]. Inert electrodes such as platinum electrodes are always used in iontophoresis. During the ionization process using inert electrodes, the PH of the anode will decrease progressively, while the PH of the cathode will increase over time [2].

The toxicity of sclera iontophoresis has also been demonstrated in clinical experiments. In one trial, corticosteroid iontophoresis was applied to the patients who had underwent a penetrating keratoplasty to alleviate the corneal graft rejection response [49]. During transscleral iontophoresis, the applicator was placed in intimate contact with the conjunctival surface, while the drug solution was in direct contact with the conjunctiva and sclera. Symptoms such as mild or minimal pain sensation were reported by approximately 50% of the patients. In most cases, transient conjunctival redness was observed immediately after iontophoresis. These signs were caused by conjunctival vascular dilation and disappeared within 24 h [49]. Intriguingly, after the iontophoresis applications, the corneal epithelium and intraocular pressure of these patients were normal, without any damage in the vitreous body, retina, and choroid till the end of the clinical trial [49]. The conjunctival damage should be ascribed to the tight connection between the applicator and the conjunctiva surface. Thus, researchers should pay more attention to the potential risks and drawbacks of iontophoresis technology.

With recent developments in iontophoresis devices, some of the aforementioned issues have been resolved. Monika Voigt et al. developed a CCI system that can induce and maintain a constant electrical field across the conjunctival epithelium barrier [50]. The CCI system produces a constant drug flow with minute negative pressure, which secures the continuous contact interface between the ocular tissue and drug solution. This system can adapt automatically to alterations in tissue impedance at the site of the return electrode. The constant current will assure a constant drug supply to the target area [41]. At the same time, Behar Cohen et al. have formulated a safety procedure for transscleral iontophoresis with current intensity smaller than 50 mA/cm^2^ [4]. Within the scope of this safety procedure, many studies did not find any evidence of inflammation or toxicity in the intraocular tissues during iontophoresis treatment [2]. However, safe current intensity exceeding the recommended range would pose a potential risk of injury. Thus far, various unwanted side effects of the iontophoresis system, including thermal disturbance, chemical insults, and mechanical damage, still exist, and impede the further application of iontophoresis. An advanced iontophoresis device with appropriate parameters is in emergent need for clinical practice.

## 6. Application of Hydrogel Iontophoresis

Mechanisms such as electrophoresis, electroosmosis, and electroporation contribute collectively to the transport enhancement during iontophoresis. It is probable that the amount of delivered drug will vary proportionally according to the applied electrical intensity. In order to attain the required drug concentration, we can either apply iontophoresis for a longer period at low current density, or for a shorter period of time at higher current density [51]. However, the increase in current density and total application time may pose potential risks to the eye architecture. Monti et al. have evaluated the electrically induced corneal damage by measuring the hydration level (HL%) of cornea tissue [52]. If a current density of 2.56 mA/cm^2^ was applied, even with only 5 min of action, the HL% increased significantly, indicating that corneal damage occurred during iontophoresis. Tim et al. investigated the effect of prolonged transscleral iontophoresis on the rabbit retina using a current of 1.5 mA [53]. The results showed that the diameter of the lesion increased significantly if the iontophoresis time increased. After a prolonged application of iontophoresis (lasted for 15 or 25 min), most of the choriocapillaris and large vessels were occluded, resulting in complete retinal necrosis. Electrolysis always occurs in the electrode chamber during iontophoresis and produces charged hydroxide (or hydrogen) ions. Due to the accumulation of charged hydroxide ions, chemical burns may occur in the areas undergoing iontophoresis [53]. Moreover, a subsequent increase in osmolality in the surrounding area may also cause cellular damage when the hydroxide ions move into the ocular tissue [53]. To this end, the hydrogel has been used as an iontophoresis buffer to avoid potential side effects.

**Figure 2:**
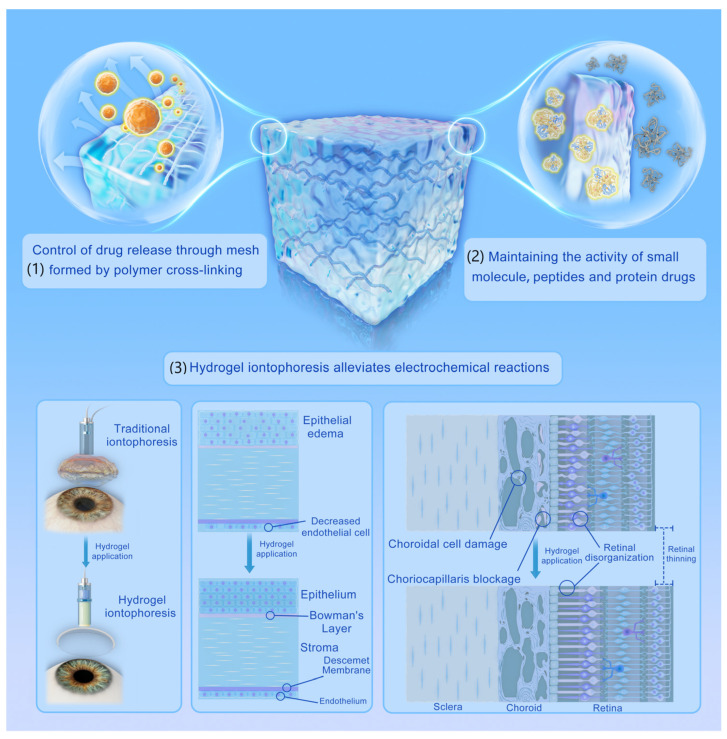
The main advantages of hydrogel iontophoresis. (**1**) The drug release mechanism of hydrogels is mainly controlled by the mesh size. Drugs with a relatively small size can diffuse rapidly through hydrogels. When the size of the drug approaches the mesh size, the rate of drug release slows down. Once the drug diameter is larger than the mesh size, it is physically encapsulated in the mesh. (**2**) The mild preparation conditions and water rich content make hydrogels an ideal material to preserve the activity of small molecule, peptides, and protein drugs over extended times. (**3**) When iontophoresis is performed on the ocular surface, the application of hydrogel can significantly alleviate corneal damage (corneal epithelial edema and decreased endothelial cell) or posterior segment tissue damage, including choroid cell damage, choriocapillaris blockage, retinal thinning and its cell disorganization.

Hydrogels have three-dimensional network structures that are cross-linked by polymers (Figure 2) [12,54]. They are produced from virtually any hydrophilic polymer through physical or chemical cross-linking [55,56,57]. Physical hydrogels can be prepared by crystallization, amphiphilic copolymers, charge interaction, hydrogen bonds, stereo-complexing, and protein interactions [58]. On the other hand, chemical hydrogels are produced through graft copolymerization, irradiation crosslinking, reactive functional groups, and enzymatic method [58]. Hydrogels contain the solid components with definite shapes, as well as the liquid components with large amounts of free water [59]. The solid phase of hydrogel is composed of a polymer usually called the mesh [55]. The size of the mesh holes depends on the linear distance between two crosslinking points (ξ). A study used the rheological analysis to demonstrate that the average molecular weight between crosslinking points (Mc) and the cross-link density (Ve) can affect the ξ of mesh size [60]. Due to the hydrophilic groups in the polymer structure, such as –OH, –CONH–, –CONH_2_–, and –SO_3_H, hydrogels can absorb and retain a large amount of water, thus endowing them with a physical similarity to biological tissues. The excellent biocompatibility and high encapsulation efficiency are two major advantages of hydrogels [61,62]. Hydrogel meshes can hold water by their hydrophilic components and release water under the drying conditions, allowing them to maintain the relatively stable elastic force [55,63]. Mechanically, hydrogels often have stiffness/Young’s modulus in the range of 0.1–100 kPa, which allows them to accommodate to various types of cells and tissues [64]. These special biological characteristics enable hydrogel to bridge the gaps between stiff electronics and biologic structures. In addition, the external stimulus, which will induce the draining or absorbing free water, can easily affect the shape and size of the hydrogels. During this process, the reactive, monomeric, and potentially polymerizable species will enter the interstice of the hydrogel matrix and occupy those empty spaces with the assistance of water [65].

There are several types of natural hydrogels, like alginate, chitosan, cellulose collagen, and gelatin, as well as synthetic hydrogels, like poly (2-hydroxyethyl methacrylate), polycaprolactone (PCL), or a biohybrid [54,65,66]. However, the poor mechanical properties such as rigidity and stretch ability of natural hydrogels limit their application, although they have good biocompatibility and biodegradability. In contrast, despite that the synthetic hydrogels have poor biological activity and unsatisfactory biocompatibility, they can withstand strong mechanical loads [54]. In the past two decades, natural hydrogels have been replaced gradually by synthetic hydrogels, since they have longer service life, stronger water absorption, and higher gel strength. More importantly, synthetic polymers typically have clear chemical structures that can be modified to produce customizable degradability and functionality [57]. Advantageously, the mild preparation conditions and high-water content make hydrogels ideal materials for preserving the activity of biologic drugs over extended periods of time [56]. Researchers synthesized hydrolytically degradable hydrogels that were composed of 4-arm PEG-vinyl sulfone (PEG-VS) and PEG-diester-dithiol cross-linkers [67]. They compared the secondary structure before and after bovine serum albumin (BSA) encapsulation using circular dichroism experimental. The researchers did not detect any significant difference or spectral shift between their spectra. They confirmed that the secondary structure of BSA was retained during the encapsulation process and subsequent release. In particular, the BSA has been released completely in the form of natural monomers.

Before hydrogels swelling, the drugs molecules in hydrogels are immobile. Once the hydrogels undergo a glass-to-rubber phase transition during swelling, these drug molecules begin to spread rapidly. The rate at which drugs are released from entrapped areas is largely dependent on the swelling level (i.e., swelling-controlled release) [68]. In addition, the mesh size of the hydrogel can be changed, which makes it possible to control artificially the release of drugs at different speeds. A recent study produced a PEG-based hydrogel with the mesh size reduced to the order of the hydrodynamic radius (about 4 nm) of BSA, which effectively immobilized the protein in the PEG network [69]. In parallel with the hydrolysis and degradation of the hydrogel network, the mesh size will increase gradually, so that BSA can be continuously released within two months. Moreover, researchers have devised an iontophoresis device based on HIC, which effectively buffer the pH change caused by EC reactions, and reduce the heating caused by electrode overvoltage [70]. Therefore, equipment assisted by hydrogel can avoid potential tissue damage caused by traditional iontophoresis equipment. In clinical practice, simple eye drops are always removed from ocular surfaces by eye blinking. However, the viscoelasticity of a hydrogel can keep them in contact with the ocular surface for a longer period of time, thereby reducing the need for repeated applications [71,72].

## 7. Developments and Application of Hydrogel in Ophthalmic Iontophoresis

**Figure 3:**
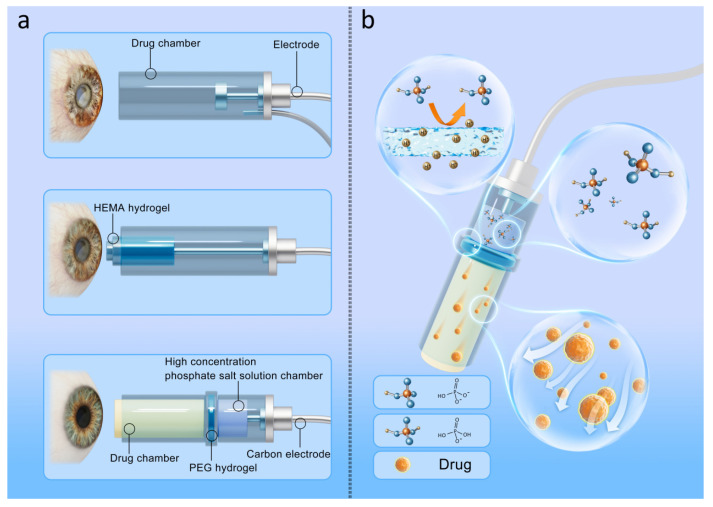
(**a**) The initial generation of iontophoresis device does not use hydrogel, which may cause mechanical damage to the patient due to the hard eye cup, while the EC reaction may cause damage to the cornea, sclera, and retina. The soft hydrogel eye cups were first utilized to alleviate the mechanical damage caused by the hard eye cups but did not solve the EC reaction caused by the iontophoresis. The third-generation modification of the ionophoresis device cleverly utilizes the role of ATPs of hydrogels to reduce the EC reaction of ionophoresis. (**b**) The PEG hydrogel acts as an aqueous two-phase separation (ATPs) between drug solution and phosphate solution. This design allows the EC reaction that occurs when the electron current is converted to ion current to occur only in the phosphate solution and not to diffuse into the drug solution. However, protons with small particle size can pass through PEG hydrogels to deliver ionic currents into the drug solution. In this way, the action of the ATPs of the hydrogels both reduces the EC reaction and allows a smooth flow of current to the drug.

Early iontophoresis used an eye cup to hold the drug solution for direct delivery [36]. This can lead to mechanical damage to the cornea or sclera during iontophoresis. Moreover, the eye cup should be sterilized before each application. Potential corneal or scleral infections may occur if the eye cup is not properly sterilized. In this context, the researchers have directed their research towards the modification of the eye cup (Figure 3a). Drossman and Frucht used disposable drug-loaded agar gels to address these issues [73,74,75]. Although the soft drug-laden agar gel can alleviate the mechanical damage caused by the eye cup, the electrical heat generated by iontophoresis process will leave some fragile agar gel on ocular surface. Therefore, researchers attempt to synthesize stable hydrogels to replace the fragile agar gels [6]. Some stable hydrogels were synthesized from hydroxyethyl methacrylate (HEMA), ethylene glycol dimethacrylate (EGDMA), and deionized water. They investigated the effects of different ratios of cross-linking agent (EGDMA) (0.5–4%) and deionized water (53–80%) in the drug content and physical properties of hydrogels. The results showed that as the percentage of deionized water in the hydrogel increased, the hydrogel would be endowed with higher water absorption and softness. On the other hand, the hardness, mechanical strength, and stability of the hydrogel will be enhanced in parallel with the increase of EGDMA percentage. Eventually, researchers propose that the hydrogel composed of 2% EGDMA, 98% HEMA (EGDMA:HEMA = 2:98) and 75% water has good water absorption and stability, and this formulation is most suitable for iontophoresis [6]. Thus far, these hydrogels have been devised for iontophoresis and widely used in animal experiments [8,9,10,11,76].

### 7.1. Application of Hydrogel in Iontophoresis

Hydrogels with proper formulation can improve the efficiency and safety of ocular iontophoresis. They have been used to transport different types of drugs or substances (such as gentamicin, methotrexate, Dex, carboplatin, charged fluorescent nanoparticles, etc.) for iontophoresis. In these experiments, the hydrogel iontophoresis system showed superior and satisfactory performance. For instance, incorporation of drugs into hydrogels permits the modulation of their release kinetics during iontophoresis experiment. This is an important advantage for the development of novel pharmaceutical formulations [77]. For instance, in the experiment conducted by Joseph Frucht-Perythe et al., the killing effect of *P. aeruginosa* was related mostly to the concentration of gentamicin. With the assistance of hydrogel iontophoresis system, it is possible to control the concentration of gentamicin in the corneal tissue by changing the current intensities, the drug concentrations within the hydrogel probe, or the duration of iontophoresis application [9]. Meanwhile, compared with the traditional iontophoretic device, the gentleness and softness of the medicated hydrogel can minimize tissue irritation and current interruption, thereby facilitating drug handling during iontophoresis [12].

#### 7.1.1. The Effectiveness and Safety of Hydrogel-Based Ionophoresis

The effectiveness of the hydrogel iontophoresis system depends on whether the drug introduced into the ocular tissue can attain the therapeutic concentration after application. In most of the experiments using the hydrogel iontophoresis system, the drug could reach the therapeutic concentration within the ocular tissue [8,10]. The drug concentrations in the eyes of the test animals were similar or even higher compared with the traditional invasive intraocular injection. For instance, researchers have used the hydrogel iontophoresis to deliver methotrexate into rabbit eyes [10]. The results showed that short-term transscleral hydrogel iontophoresis of methotrexate could produce therapeutic drug concentration in the ocular tissues (except the vitreous) and maintain the level for several hours. Among them, the therapeutic drug level was maintained in the sclera and retina for at least 8 h, and in the aqueous humor for 2 h. The concentration of methotrexate in the retina and uvea tissue was sufficient for treating inflammatory diseases, since the required therapeutic level of methotrexate is relatively lower in pharmaceutics (0.2 µg/mL) [10]. Furthermore, a recent pharmacokinetics study showed that a single application of 1 mA iontophoresis using a gentamicin-containing hydrogel probe could attain high bactericidal concentration of gentamicin in the corneal tissue (363 mg/g); 8 h after iontophoresis, the drug concentration in the cornea remained bactericidal with 24.9 mg/g. This concentration was similar to the drug concentration (22.7 mg/mL) using topical 1.4% gentamicin solution every 5 min for 1 h [8].

The safety of the hydrogel iontophoresis system depends on whether the structure and biomorphic feature of eyeballs are normal after iontophoresis. Thus far, the safety of hydrogel has been reported in multiple experiments [8,9,13,17,76]. For instance, the typical hydrogel iontophoretic system is a battery-operated portable device. Light equipment weight and biological compatibility of hydrogel can help to reduce the damage caused by the applicator contact. Furthermore, hydrogel can reduce tissue stimulation and current interruption, and allow the adjustment of pharmacokinetics by changing the characteristics of hydrogel component. In general, hydrogel has indeed improved the efficiency and safety of iontophoresis experiment.

#### 7.1.2. The Concentration and Distribution of Drugs in Hydrogel Iontophoresis

During the application of hydrogel iontophoresis, influential factors such as the current duration, current intensity, drug concentration in the hydrogel, contact area between the hydrogel and ocular surface, would collectively determine the drug penetrability. The molecular charge, the molecular weight, and the liquid solubility, on the other hand, would determine the drug’s persistence. All of these factors can affect the concentration and distribution of drugs in the eye tissue. The effects of current duration, current intensity, and drug amount in the hydrogel on drug penetration have been studied in many iontophoresis experiments. Joseph Frucht-Pery et al. found that increasing the current intensity or prolonging the duration of iontophoresis enhance the penetrating ability of gentamicin into the cornea. Once the drug concentration in the hydrogel is determined, the current intensity will affect the drug penetration rather than the apply duration. Moreover, the researchers have verified the clinical significance of the hydrogel iontophoresis system in treating microbial keratitis, as it would allow the controlled release of a drug into a small and localized infected area [10]. Esther Eljarrat-Binstock and Abraham J. Domb also studied the effects of altered current density and drug dosage on the efficiency of hydrogel iontophoresis [10]. In the transscleral iontophoresis experiments of methotrexate, the researcher applied a weak current with small drug dosage (a current density of 1.6 mA/cm^2^ with the hydrogel drug load of 2 mg) and compared the efficiency with that of a high current with large drug dosage (a current density of 5 mA/cm^2^ with the hydrogel drug load of 10 mg). After increasing the current density and drug dosage, a twice fold-higher concentration was achieved in the vitreous; in the retina and sclera, 8 to 20 folds higher concentrations were attained as well [10]. Similar phenomena have also been reported in another experiment using gentamicin to treat pseudomonas keratitis in rabbit corneas. After iontophoretic treatment with a high current at 0.5 mA, the logarithmic value of Pseudomonas colony forming units (CFUs) was 2.96 ± 0.45. On the other hand, after lower current (0.2 mA) iontophoretic treatment, the logarithmic value of Pseudomonas CFUs was significantly larger (5.25 ± 0.54) [9]. Furthermore, the influence of the contact area between hydrogel and ocular surface has also been studied. Researchers used two types of hydrogels with different contact surface areas for iontophoresis experiments. For the first experimental group, they used rectangular hydrogels of 1.2 cm length and 0.5 cm width and height, resulting in a surface area of 0.6 cm^2^. For other experimental groups, cylindrical hydrogels of 0.5 cm diameter and height were used, resulting in a contact surface area of 0.2 cm^2^. These results suggest that utilization of small cylindrical hydrogels with low current intensity (1 mA) would be more effective for delivering ocular drugs than using the rectangular hydrogels with higher intensity [12]. In view of this evidence, researchers assume that the small hydrogel surface will contribute to a better compatibility with the ocular tissue, and result in a better diffusion efficiency of drugs [12].

In the hydrogel iontophoresis system, the penetrating efficiency of drugs is also closely correlated with the physical and chemical properties of drugs: (1) Molecular charge of drugs; Esther Eljarrat-Binstock et al. have used a hydrogel iontophoresis system to deliver charged fluorescent nanoparticles into rabbit eyes. They compared the permeation results of positive and negative nanoparticles. After applying a low current of 1.5 mA for 5 min, negative charged nanoparticles were electrically repulsed into the anterior and posterior tissues within the first 30 min. Then the negative charged nanoparticles migrated from the outer ocular tissues to the inner ocular tissues, such as the retina and choroid. However, the induction effect of positively charged nanoparticles with low current was more prominent than that of negatively charged nanoparticles. Iontophoresis technology enhanced the penetration of positive nanoparticles into external and internal tissues. However, when drug molecules were passively diffused, most positive nanoparticles only stayed within the corneal tissue [13]. Accordingly, the positive particle seems to be a better candidate for being a drug carrier than the negatively charged particle in hydrogel iontophoretic [13]. This preference is in accordance with the results of another literature, in which the positive charges participate in the electrostatic interaction with the negatively charged corneal, and, thus, increasing the concentration and residence time of the associated drug [13,78]. (2) Diffusion properties of drugs; In a hydrogel iontophoresis experiments, the carboplatin concentration achieved 18.88 ± 3.48 (the mean concentration ± SD) in sclera under the treated area, after applying a 5 mA/cm^2^ current for up to 10 min [12]. The researchers propose that the degree of drug ionization and the drug diffusion capacity also act as an influencing factor of the iontophoresis results.

### 7.2. Updates of Hydrogel Probes in Iontophoresis

Although numerous experiments have confirmed that the initial generation of drug-carrying hydrogels could improve the performance of iontophoretic devices to some extent, the most fundamental paradox between the EC reaction caused by high currents and the inability of small currents to deliver effectively drugs has not been resolved absolutely. The basic principle of iontophoresis is that an electric current drives the drug particles and widens the gap of the ocular barrier, making it easier for the drug particles to penetrate the ocular barrier and reach the lesion [17,30]. The electron currents from the ionophoresis generator can be converted to ion currents when they reach the drug solution. The conversion process of electron currents to ion currents requires EC reactions (including the changes in pH caused by electrolysis and the generation of Joule heat caused by large resistance in the drug solution) [79,80,81]. For instance, adverse effects were reported by Esther Eljarrat-Binstock when using HEMA for iontophoresis. In that case, minor reversible epithelial defects and stromal edema were found 5 min after the application of a single iontophoretic treatment at a current intensity of 0.5–1 mA for 60–120 s. However, the damage disappeared after 8 h after treatment [6]. Moreover, in another iontophoresis experiment (at a current intensity 4.0 mA), conducted on healthy volunteers, two out of four subjects reported a burning sensation in the application area, and these symptoms disappeared within 22 h after treatment [82].

In this context, a novel iontophoretic device dubbed HIC has been devised to resolve these safety problems [5]. This device’s eye cup is composed of three parts. The tail part consists of saturated phosphate salt solution with high conductivity, which is directly connected with the electrode. The design of the tail allows the ion current conversion to occur directly in the phosphate solution. The high conductivity and buffering effect of phosphate not only reduce the generation of Joule heat, but also balance the changes of pH. The head of the eye cup consists of a drug solution. The body part of the eye cup is composed of PEG hydrogel that separates the drug solution from the phosphate solution. PEG hydrogel acts as an aqueous two-phase separation (ATPs) between drug solution and phosphate solution (Figure 3b). On the other hand, the ionic current can still pass through the PEG hydrogel during iontophoresis. Researchers have compared this hydrogel-based device with a conventional iontophoretic device using an experimental model of rabbit eye. They found that the safe current density of the hydrogel-based device was as high as 87 mA cm^−2^ [5]. In contrast, the maximum current density of conventional devise usually does not exceed 7.5 mA cm^−2^ [30]. For instance, the conventional ocular iontophoresis with a current density of 15 mA cm^−2^ could result in significant ocular burns [83]. These findings indicate that HIC has a safe current density that is approximately ten times higher than conventional iontophoretic devices. Furthermore, an efficiency study also showed that iontophoresis using high current density was 300 times more efficient than conventional low current iontophoresis to deliver the same concentration of nanoparticles or macromolecular drugs [5] (Table 2).

## 8. Conclusions

The complex physiology and anatomy of the eye presents a huge problem for the treatment of ocular diseases. In order to deliver drugs across biological barriers and reach therapeutic concentrations within the lesions, the iontophoresis technique, which uses an electric field created by a tiny electric current to drive charged drug particles, has been devised in the past decades. The development of iontophoresis technology has cast lights into the zone of non-invasive therapeutics for ocular diseases. In order to enhance delivery efficiency, reduce the EC response, and improve patient compliance during treatment, hydrogel has been incorporated into the iontophoretic devices. Hydrogel has a three-dimensional mesh structure with soft texture and predictable shape. Advantageously, the absorption, storage, and isolation properties of hydrogel can compensate for some shortcomings of conventional iontophoresis. Accumulating evidence have verified the safety and effectiveness of the hydrogel iontophoresis devise across a range of ophthalmic indications. In this paper, we have introduced recent improvements in the iontophoresis technique and summarized the performance of hydrogel-based iontophoretic devices in both animal experiments and clinical trials. We also compared the efficiency of hydrogel-based iontophoresis with conventional iontophoretic devices and proposed that hydrogel might act as an essential component of iontophoretic devices in ophthalmic practice. The application of hydrogel component depends on the future developments in chemistry and materials science. Thus far, hydrogels only play a single role in the iontophoresis technique, such as reducing the EC reaction or mitigating the mechanical injury caused by the eye cup during iontophoresis. Existing hydrogel devices cannot combine the marvelous advantages in terms of effectiveness and safety. It is expected that further developments in hydrogel design and production would facilitate the utilization of iontophoresis in ophthalmic practice. The standard of ophthalmic iontophoresis technology has been established gradually. In particular, the hydrogel ionic circuit system has a sophisticated application standard with simple operation procedures. This generation of hydrogel iontophoretic device could be easily used by ophthalmologists after systematic training.

The structure characteristics of solid and liquid two-phase three-dimensional networks, as well as the water absorption characteristics caused by hydrophilic groups in polymers, are the main advantages of hydrogels. This makes the hydrogel itself have the ability to carry and release drug solutions. However, the hydrogel materials should be improved for use in ocular iontophoresis devices. In order to meet the drug delivery requirements of different iontophoresis experiments, hydrogel materials should be able to deliver drugs with diverse features and have high delivery efficiency and controllable release rate. This requires better controllability and modifiability of hydrogels. Additionally, hydrogels used as applicators in iontophoresis devices should have good biocompatibility, mechanical properties, and stability in order to adapt to the ocular environment. Most importantly, hydrogels are able to alleviate ocular tissue damage caused by changes in pH and temperature. Scientific researchers have repeatedly explored and proved that the synthetic hydrogel has a clearer chemical structure than the natural hydrogel. By using different synthetic polymers and mixing their proportions, the hydrogel can be modified to produce customizable the swelling level and mesh size. At the same time, these procedures can also restructure the biocompatibility, mechanical strength, and stability of the hydrogel to adapt to different iontophoresis devices. An enhanced current can improve the delivery efficiency of macromolecular drugs. However, the eye damage caused by a high current is more serious, which is difficult to solve by changing the composition of the hydrogel alone. In order to break the limitation of eye injury on the current intensity, it is necessary to develop new materials combined with hydrogels. For example, in the PGE hydrogel device, the damage of a high current to eye tissue is mainly alleviated by high-concentration phosphate salt solutions. What the hydrogel needs to do is to provide sufficient mechanical strength to support the weight of high-concentration phosphate salt solutions in the device, and to form ATPS to effectively isolate phosphate solution and drug solution, while maintaining its own stability and conductivity. In addition, the mild manufacturing conditions and long service life of hydrogels, as well as the the hydrogel device being portable and simple to operate, are important conditions for the wide application of a hydrogel iontophoresis device in experiments and clinical treatment.

## Figures and Tables

**Table 1:** Experimental studies of the application of hydrogel in iontophoresis.

Hydro Material	Delivery of Substances	Route	Experimental Purpose	Current Intensity and Application Time	The Distribution Pattern of Delivered Substances in the Eye	Iontophoresis Results	Reference
Hydroxyethyl methacrylate gel discs	Gentamicin	Transcorneal	The effect of gentamicin penetrating rabbit cornea using hydrogel iontophoresis.	0, 0.1, 0.3, and 0.6 mA for 10 and 60 s, respectively	Very small or no concentrations of the drug were discovered in the anterior chambers of rabbits treated with current intensity of 0.1 mA.	Achieved therapeutic concentrations of gentamicin into the cornea	[7]
		Transcorneal	To evaluate the transcorneal iontophoresis of gentamicin by drug carrying water gel probe.	1 mA for 60 s	Peak gentamicin concentrations in the cornea and in the aqueous humor were reached after 2 h. The peak gentamicin concentrations after a single iontophoresis treatment were 12 to 15 times higher than those obtained by topical eye drop instillation.	The medication in the cornea can maintain treatment levels for more than 8 h	[8]
		Transcorneal	The effects of hydrogel probe in the application of low current iontophoresis to deliver gentamicin to the eyes.	1 mA for 240 s, 0.5 and 1 mA for 60 and 120 s	High gentamicin concentrations were found in the retina and sclera 4 h after the iontophoretic. Meanwhile, there were lower concentrations in the cornea, aqueous humor, and vitreous.	High drug concentration can be obtained at posterior segments of the eye; 0.5 and 1 mA for 60 and 120 s is safe in terms of current protocols.	[6]
		Transcorneal	The efficacy of gentamicin loaded hydrogel iontophoresis system in the treatment of pseudomonas keratitis in rabbits.	0.5 and 0.2 mA for 60 s, repeat it three times at intervals of 3.5 h	None	A short time iontophoretic treatment using gentamicin loaded hydrogels has potential clinical value in treating corneal infections.	[9]
	Methotrexate	Transscleral	To evaluate the permeability and distribution of methotrexate in the ocular structure after hydrogel iontophoresis.	Low density: 1.0 mA for 2 min; High density: 1.0 mA for 5 min	After iontophoresis with low current density, the therapeutic drug levels were maintained at the sclera and retina for at least 8 h, and the aqueous humor for 2 h. When the current density was increased, the concentration of the vitreous body doubled, and the concentration of the retina and sclera increased by 8–20 times.	A potential clinical value in treating ocular inflammatory diseases and intraocular lymphoma.	[10]
	Dexamethasone	Transcleral, transcorneal	To evaluate the penetration of dexamethasone into the eyes after hydrogel iontophoresis.	1 mA for 1 or 4 min	After 1 min of single corneal iontophoresis, the level of dexamethasone in the cornea was 30 times higher than that of eye drops. After 4 min of transscleral iontophoresis, high concentrations of drugs were obtained in the anterior and posterior segments of the eye.	A short time current non-invasive iontophoretic treatment using dexamethasone-loaded hydrogels has potentials in increasing drug penetration to the anterior and posterior segments.	[11]
	Carboplatin	Transsclera	The penetration efficiency of carboplatin in the hydrogel iontophoresis in vivo and in vitro.	0, 1, and 3 mA current for 10 min	Higher levels of drugs were found in the sclera and retina, while lower levels were found in eye fluids.	Passive carboplatin diffusion from loaded hydrogels inserted in the lower cul-de-sac may be a potential clinical treatmentfor intraocular retinoblastoma.	[12]
	Positively or negatively charged fluorescent nanoparticles	Transscleral, Transcorneal	The penetration efficiency of charged fluorescent nanoparticles into rabbit eyes was evaluated using hydrogel iontophoresis.	1.5 mA for 5 min	The positively charged particles demonstrated better penetration abilities into inner ocular tissues compared to the negatively charged particles.	The use of charged nanoparticles as ion carriers for drug delivery can provide extended treatment activity beyond 12 h after treatment.	[13]
PEG hydrogel	Dextran-40kDa	Transscleral	To determine whether the high-intensity ion current safely applied by our HIC-based device could be enhanced by hydrogel iontophoresis.	100 mA (87 mA cm^−2^) for 20 min	High-intensity iontophoresis increased the amount of dextran delivered into the vitreous by 122 times. A significant concentration of dextran presented in all tissue segments from conjunctive/sclera to vitreous.	Compared to traditional iontophoresis devices, the hydrogels device achieved over 100-time enhancements. High-intensity currents also increased the drug penetration into deeper ocular tissues.	[5]
	Bevacizumab	Transscleral		100 mA (87 mA cm^−2^) for 20 min	Bevacizumab was delivered into the vitreous, similar to the amount delivered by intravitreal injection. A diffusion-like concentration gradient was established from conjunctiva to vitreous.		

Abbreviations: PEG hydrogel: polyethylene glycol hydrogel. Composition of hydrogels: PEG hydrogel consists of 10% *w*/*w* poly (ethylene glycol) dimethacrylate (PEGDMA, molecular weight (MW): 8000) and 5% *w*/*w* poly (ethylene glycol) diacrylate (PEGDA, MW: 700). Hydroxyethyl methacrylate gel discs consist of HEMA (97% pure), EDGMA (98% pure), deionized water_,_ 2% sodium persulfate Na_2_S_2_O_8_, 2% sodium metabisulfite Na_2_S_2_O_5_, and 2% ammonium ferrous sulfate Fe(NH_4_)_2_(SO_4_)_2_.

**Table 2:** Comparison of conventional iontophoresis devices and hydrogel-based iontophoresis devices.

Development of Hydrogels in Iontophoresis Devices	Experimental Animal	Route	Maximum Current Density (or Current) and Application Time	Ocular Damage	Cause of Damage	Hydrogel Synthesis Method	The Function of Hydrogel	Advantages over Conventional Iontophoresis Devices	Reference
Conventional iontophoresis device	Rats	Transcorneal	2.11 mA/cm^2^ for 4 or 10 min	No	None	None	None	None	[40]
	Rabbits	Transscleral	4 mA/cm^2^ for 4 or 10 min	No	None				[4]
	Rabbits	Transscleral	5 mA/cm^2^ for 10 min	A slight injection of conjunctiva	The negative pressure applied on the eye during CCI, mechanical damage				[38]
	Rabbits	Transscleral	5 mA/cm^2^ for 10 min	A slight injection of conjunctiva	The negative pressure applied on the eye during CCI, mechanical damage				[41]
	Rats	Transscleral	1.2 mA/cm^2^ for 4 min	Transient conjunctival vasodilatation	The negative pressure applied on the eye during CCI, mechanical damage				[36]
	Mice	Transscleral, Transcorneal	greater than 5.14 mA/cm^2^ for 2 to 5 min	Slight damage to the corneal epithelium and/or minor scleral burn-like yellowing	EC reaction				[37]
	Rabbits	Transscleral	5 mA/cm^2^ for 20 min	A slight injection of conjunctiva and transient swelling of the conjunctiva	EC reaction or the negative pressure applied on the eye during CCI, mechanical damage				[39]
Application of first-generation hydrogel	Rabbits	Transcorneal	0.6 mA for 10 and 60 s	No	None	Polymerize at room temperature, lyophilization dehydrated, hydrate	Soft, slow-release, drug-loaded, reduces eye infections	Reduces conjunctival injection and swelling caused by negative pressure or mechanical damage due to the eye cup during iontophoresis	[7]
	Rabbits	Transcorneal	5.1 mA/cm^2^ for 60 s	No	None				[8]
	Rabbits	Transcorneal, Transscleral	1 mA for 240 s	Mild reversible effect including stromal edema and epithelial defects	EC reaction				[6]
	Rabbits	Transcorneal	0.5 mA for 60 s	No	None				[9]
	Rabbits	Transscleral	5 mA/cm^2^ for 5 min	No	None				[10]
	Rabbits	Transcleral, transcorneal	5.1 mA/cm^2^ for 1 or 4 min	No	None				[11]
	Rabbits	Transsclera	5 mA/cm^2^ for 10 min	No	None				[12]
	Rabbits	Transscleral, Transcorneal	6 mA/cm^2^ for 5 min	No	None				[13]
Application of second-generation hydrogel	Rabbits	Transscleral	87 mA cm^−2^ for 20 min	No	None	photocrosslinking	ATPs	Reduce damage caused by EC reaction during iontophoresis	[5]
	Rabbits	Transscleral		No	None				

## Data Availability

Data sharing not applicable.
